# Looking Forward: Evaluating Management Scenarios for an Isolated Amphibian Population in a Dynamic Coastal Environment

**DOI:** 10.1002/ece3.72598

**Published:** 2026-01-12

**Authors:** Alex Callen, Heather Maher, John Gould, Matt W. Hayward, Michael Mahony, Gabriel C. Rau, Samantha Sanders, Sarah Stock, Kate Tunstill, Darren M. Southwell

**Affiliations:** ^1^ Centre for Conservation Science, School of Environmental and Life Science University of Newcastle Callaghan New South Wales Australia; ^2^ School of Environmental and Life Sciences University of Newcastle Callaghan New South Wales Australia

**Keywords:** amphibian, fragmentation, genetic diversity, *Litoria aurea*, population persistence, population viability analysis

## Abstract

Urbanisation impacts biodiversity in coastal ecosystems. Conservation management is needed to improve species persistence in such areas where populations have become small and fragmented. We conducted a population viability analysis to compare management scenarios for an isolated population of the threatened green and golden bell frog ( *Litoria aurea* ) in a peri‐urban area of coastal south‐east Australia. The breeding population occupies a single wetland that is hydrologically connected to an intermittently open lagoon. The lagoon is periodically drained to reduce flood risk to residential areas, influencing the reproductive output of the population. We combined estimates of population size, demographics, dispersal and genetic diversity to compare the relative probability of local extinction over a 25‐year forecast window under four management scenarios: (1) lagoon draining at historic rates (status quo); (2) halving the incidence of lagoon draining; (3) creating new breeding habitat; and (4) supplementing the population. Our modelling predicted that the population had a 60% probability of extinction under the status quo scenario, while halving the frequency of lagoon draining or creating a new but hydrologically distinct wetland nearby reduced the probability of extinction to 6%. Predictions of population size at the end of the forecast period never reached zero when 10 adults were supplementing the population each year. Our analysis suggested that this *L. aurea* population will likely go extinct if the current frequency of lagoon draining continues. We believed the most cost‐effective strategy to improve the persistence of the population over a 25‐year management horizon is to reduce how often the lagoon is drained so that sufficient water remains in the wetland to support egg and tadpole survival. We highlighted that artificially manipulating the hydrology of coastal environments to reduce flood risk can compromise the persistence of hydrology‐dependent species.

## Graphical abstract

Amphibians in coastal ecosystems risk local extirpation, particularly when isolated.

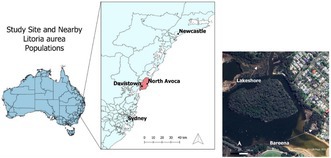

## 1 Introduction

Habitat clearing and modification for urban development has reduced once widespread species to small, isolated populations in highly fragmented landscapes (Fischer and Lindenmayer 2007). This is especially true in coastal ecosystems, with greater than 50% of these areas degraded as a result of high human activity (Williams et al. 2022). Yet, coastal systems provide many important ecosystem services such as coastal protection, erosion control and improved fishery productivity (Barbier et al. 2011; Costanza et al. 2014). As populations become fragmented, they are increasingly vulnerable to further declines due to stochastic processes in their demography (e.g., larval survival), environment (e.g., bushfires, disease outbreaks) and genetic diversity (Lacy et al. 2024; Carley et al. 2022; Cheptou et al. 2017). Implementing conservation actions is thus often necessary for species in decline or at risk of extinction; however, it can be difficult knowing a priori which management action will be most effective in the long term (Ludwig et al. 2001).

Population viability analysis (PVA) is a tool commonly used in conservation science to predict the extinction risk of small populations and to compare the influence of management alternatives (Akcakaya and Ginzburg 1991; Lacy 1993; Wintle et al. 2005). It is a modelling approach that combines information on the ecology, life history, dispersal rates and initial population size of target species to predict the likely population trajectory over a pre‐specified time horizon (Akcakaya and Ginzburg 1991). PVA is particularly relevant to small populations because it can account for genetic processes, such as inbreeding depression, as well as demographic and environmental stochasticity by sampling model parameters (e.g., survival, fecundity rates) from probability distributions (Lacy 1993). As such, PVA has estimated extinction risk and informed conservation management decisions for a range of taxa including, birds, plants, mammals, amphibians and fish (Akçakaya and Sjögren‐Gulve 2000; Reed et al. 2002).

Amphibians have undergone rapid and extensive decline over the past 50 years and are considered the most threatened class of vertebrate on the planet (Nowakowski et al. 2017; Scheele et al. 2019; Luedtke et al. 2023). A range of threatening processes has contributed to their decline, including habitat loss and fragmentation, infectious disease, competition with invasive species and climate change (Collins and Storfer 2003; Green 2003; Simpkins et al. 2022). Although progress has been made uncovering the causes and extent of such declines, many amphibian species have already been reduced to small, fragmented populations, particularly in highly modified environments such as the coastal zone (Scheele et al. 2017). A critical first step to their conservation is estimating extinction risk and identifying management strategies that might increase the probability of persistence.

The threatened green and golden bell frog ( *Litoria aurea* ) occupies less than 10% of its known historical range, having once been common and widespread across south‐east Australia (Pyke and White 2001; Hamer and Mahony 2007; White and Pyke 2008). It is now mostly confined to small, highly fragmented populations along Australia's densely populated south‐east coast, often in highly modified and contaminated coastal environments (Pyke and White 2001; Pickett et al. 2016). Key drivers of decline include: (1) infection with *Batrachochytrium dendrobatidis*, which increases mortality; (2) predation of eggs and tadpoles by the invasive mosquito fish (*Gambusia* sp); and (3) habitat loss and fragmentation, which limits dispersal between populations leading to low genetic diversity. Thus, identifying management strategies that are context‐specific and have the highest chance of improving population persistence is important for conservation of the species. Such actions may include improving the quality of existing habitat, creating new habitat, assisting dispersal between patches and population supplementation (Southwell et al. 2016).

In this study, we conducted a PVA to assess extinction risk and the relative effectiveness of management scenarios for an isolated population of *L. aurea* in north Avoca, south‐east Australia. This population occupies a freshwater wetland characterised by a single waterbody that is hydrologically connected to a coastal lagoon. The coastal lagoon belongs to a group of waterbodies described as ICOLLS (intermittently closed and open lake and lagoons). ICOLLs drain naturally to the ocean when weather conditions cause a breach or opening of the beach‐berm; for example, as the result of catchment flows from heavy rainfall events, storm scenarios and tide cycles. However, ICOLLs in peri‐urban areas are often artificially drained by management authorities to reduce flood risk to residential areas in the surrounding catchment. In the case of the *L. aurea* population in North Avoca, artificially draining the Avoca Lagoon also drains water from the freshwater wetland because they are hydrologically connected. Artificially draining the lagoon can therefore destroy *L. aurea* eggs or tadpoles in the wetland and remove the opportunity for further breeding in that season. Complete water loss in the wetland can lead to mass mortality of eggs and tadpoles and no recruitment into the population in that breeding season (Pickett et al. 2014).

In our PVA, we combined estimates of current population size, demographics, dispersal rates and genetic diversity to compare the relative probability of local extinction over a 25‐year forecast window. We compared four alternative management scenarios: (1) a ‘status quo’ scenario, where the lagoon continues to be drained at historical rates and no other management strategies are implemented; (2) the incidence of lagoon draining is halved; (3) new breeding habitat is created adjacent to the existing wetland; and (4) the existing wetland is supplemented with 10 individuals with high genetic diversity each year. In addition, we conducted a sensitivity analysis of key model parameters to explore their impact on predictions. The results of our PVA provide the first assessment of the extinction risk of a stronghold population of *L. aurea*. Our sensitivity analysis also identifies the relative contribution of ecological processes and important life history parameters on population dynamics. Understanding these processes is crucial to identifying management actions that have the lowest relative probability of local extinction.

## 2 Methods

### 2.1 Study Species

**FIGURE 1:**
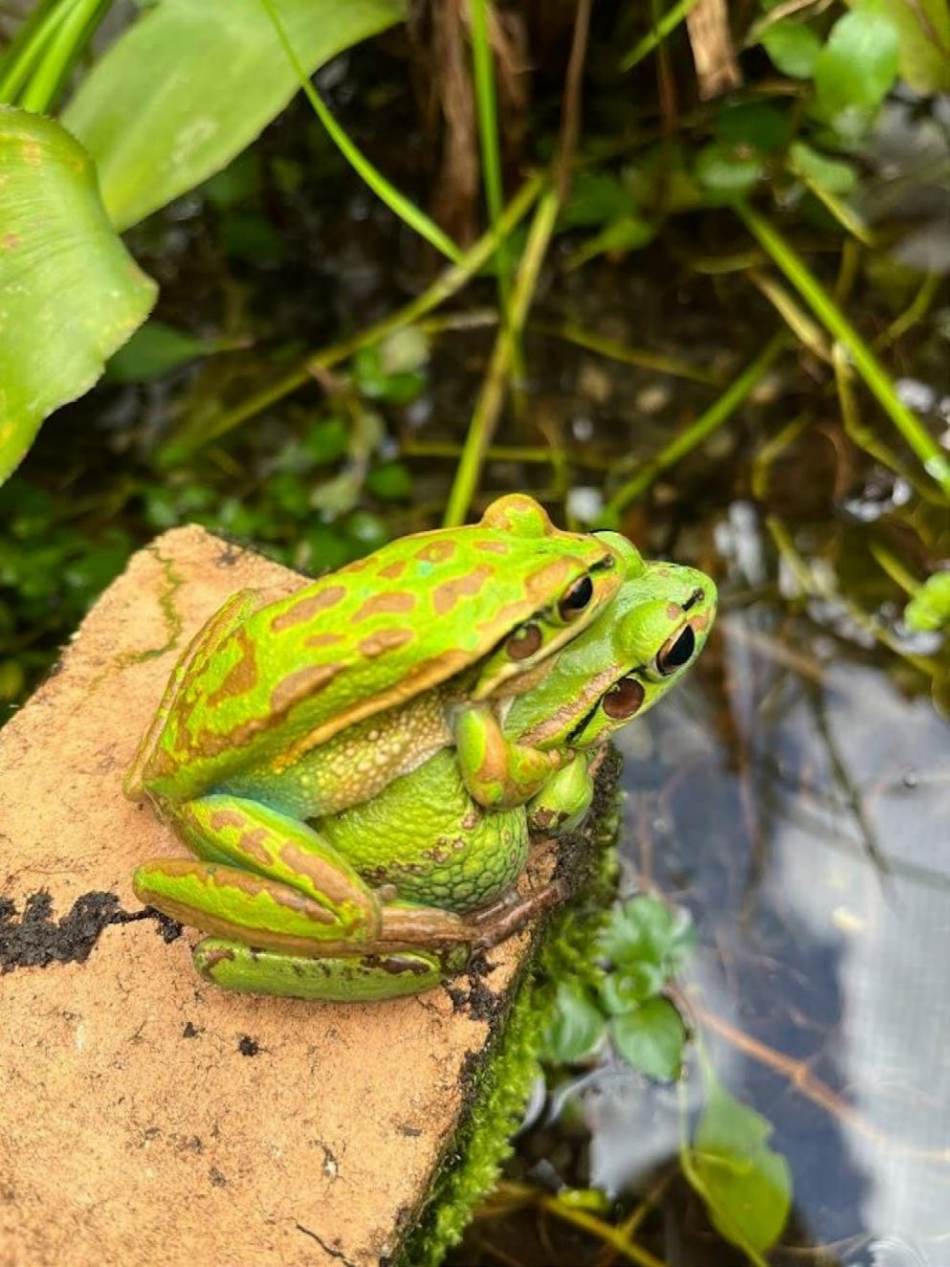
The green and golden bell frog ( *Litoria aurea* ) is listed as Near Threatened on the IUCN (International Union for Conservation of Nature) Red List of Threatened Species (IUCN 2022) and Vulnerable in Australia under the Commonwealth *Environment Protection and Biodiversity Conservation Act* (1999), having undergone a 90% reduction in its historical range. It is vulnerable to a range of threats, including habitat loss, fragmentation, predation by invasive aquatic species, and disease (*B. dendrobatidis*) and has now largely contracted to small, isolated populations along the coast. Photograph taken by Alex Callen.

The green and golden bell frog ( *Litoria aurea* ) (Figure 1) is a large species of frog with green, gold and coppery colouring. They are semi‐aquatic, typically inhabiting still or very slow‐moving shallow water bodies (< 1 m deep) exposed to sunlight (Pyke and White 2001), with individuals capable of long‐distance dispersal greater than 1 km (Hamer et al. 2008). The species breeds intermittently during Austral spring and summer (beginning of September until the end of February) with peaks in breeding activity immediately following heavy rainfall in this period. Breeding occurs in permanent, seasonally ephemeral and newly charged waterbodies and most tadpoles metamorphose within 3 months, although some can take up to 10 months (Klop‐Toker et al. 2021). Adults are tolerant of a broad range of waterbody conditions (Gould, Beranek, et al. 2024; Gould, Callen, et al. 2024).

Despite being the most fecund amphibian in Australia (Pyke and White 2001; Hamer and Mahony 2007; Pickett et al. 2016), survival to sexual maturity is extremely low (Pickett et al. 2014), and the species is vulnerable to a range of threats, including habitat loss, fragmentation, predation by invasive aquatic species, and disease (*B. dendrobatidis*). Due to a 90% reduction in its historical range, *L. aurea* populations are now largely contracted to small, isolated populations along the coast, warranting its listing as Near Threatened on the IUCN (International Union for Conservation of Nature) Red List of Threatened Species (IUCN 2022) and Vulnerable in Australia under the Commonwealth *Environment Protection and Biodiversity Conservation Act* (1999). The species' response to hydroperiod and salinity (Callen et al. 2023) is thus important for a species whose last remaining populations are confined to the dynamic coastal zone.

### 2.2 Study Site

**FIGURE 2:**
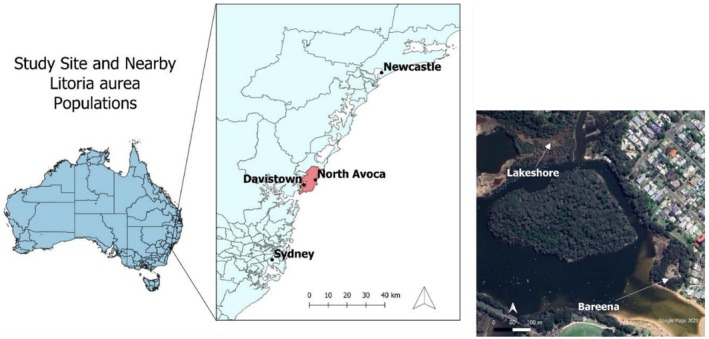
Location of the Bareena *L. aurea* population relative to its nearest population (Davistown) and two nearest major populations at Kooragang Island (Newcastle) and Sydney Olympic Park (Sydney).

The North Avoca *L. aurea* population occupies a coastal freshwater wetland (hereafter referred to as Bareena wetland) adjacent to and hydrologically connected to Avoca Lagoon (hereafter referred to as the Lagoon) in New South Wales, Australia (Figure 2). The wetland is characterised by one single, rectangular‐shaped waterbody of approximately 4000 m^2^ when fully charged. It comprises a habitat mosaic of open water, emergent reeds and *Casuarina* and *Melaleuca* forest. The wetland is separated from the lagoon by an earthen levee that restricts surface flows up to 1.45 mAHD (Palombi et al. 2025). Groundwater exchange between the lagoon and wetland has also been recently confirmed (Wainwright and Nevell 2022).

The Lagoon is 97 ha in size and supports a suite of native environments, including five endangered ecological ecosystems and coastal freshwater wetlands. It drains a catchment of 1187 ha comprising 46% rural/residential land, 9% waterways and 45% forest. The Lagoon floods during heavy rainfall due to the relatively small catchment size and surrounding land use, posing a risk to residential housing in low areas. When the Lagoon empties, the groundwater table drops and water in the wetland drains too, limiting the amount of water available for breeding habitat and reproductive output. Once the Lagoon has opened it receives ocean inflows that may also increase the salinity of the wetland (Wainwright and Nevell 2022).

### 2.3 Population Viability Analysis

#### 2.3.1 Life History Parameters

We conducted a PVA for the North Avoca *L. aurea* population using the software Vortex Version 10 (Lacy et al. 2021). We set the duration of each year in days to 365 and defined the following age classes: Female 0–1 years, Female 1–2 years, Female 2+ years, Male 0–1 years and Male 1+ years, coinciding with major events in the life cycle (eggs, tadpoles, metamorphlings and reproductively mature adults, accounting for sexual dimorphism) (Pyke and White 2001). The 0–1 year category refers to the egg, tadpole and metamorphling life stage. We assumed a sex ratio of 50:50 (Pickett et al. 2012) and set the lifespan to 10 years as this is roughly what has been observed in captivity (Pyke and White 2001). We set the initial population size to 350 individuals (males and females) based on mark‐recapture studies conducted in 2023 and a POPAN analysis (Appendix 1). We assumed a carrying capacity of 1000 individuals given the wetland is unlikely to support more than three times the current population size.

We set the reproductive system to polygynous (one male can mate with multiple females) and the mean clutch size to 5248 eggs (SD = 1148.1) based on previous enumeration of 27 egg clutches (Christy 2001). While fertilisation rates are high (91%–94%, Christy 2001), only 2–11 offspring survive to reproductive maturity per breeding female (Pickett et al. 2014). We therefore set mortality of 0–1 year‐olds to 99.9% (including the egg, tadpole and metamorph stages) to achieve this level of recruitment. We set adult mortality to 71% as derived from annual survival estimates of *L. aurea* at Sydney Olympic Park (Pickett et al. 2016). A full set of life history values is presented in Table 1.

**TABLE 1:** Model parameters used to create the population viability model for the Bareena *L. aurea* population in Vortex.

Category	Parameter	Value	Sources
Scenario settings	No. of iterations No. of years Extinction definition	500 25 years Only 1 sex remaining	
Reproductive system	Mating system Age of first offspring (female) Age of first offspring (male) Max lifespan Max no. of broods per year Max no. of progeny per brood % Males at birth Max age of reproduction Percent females breeding	Polygynous 2 years 1 year 10 years 1 8216 50% 10 years 100%	Hamer and Mahony (2007) Pyke and White (2001) Christy (2001) Christy (2001) Pickett et al. (2012)
Reproductive rates	Fecundity	5248 (SD = 1148)	Christy (2001)
Mortality rates	0–1 years Female (1–2 years) Male (1+ years) Female (2+ years)	99.9% (SD = 0.02) 71% (SD = 0.001) 71% (SD = 0.001) 71% (SD = 0.001)	Pickett et al. (2014) Pickett et al. (2016)
Catastrophes	Catastrophe type Frequency of occurrence Effect on reproductive rate Survival rate	Lagoon draining 82% 30% reduction 1	Davis et al. (2019)
Population	Initial population size Carrying capacity	350 1000	POPAN analysis from Mark Recapture data Appendix 1
Genetics	Allele frequencies	100 alleles, 2 loci	This study

#### 2.3.2 Lagoon Draining

We modelled draining of the Lagoon as a catastrophic event in Vortex (Lacy et al. 2021). We obtained records of draining events over the last 47 years from Central Coast Council and determined that there is an 82% chance of a draining event in any given breeding season (September—March). While lagoon draining is not consistent as it is tightly linked to climatic patterns, a limit of the catastrophe feature in Vortex is that the impact is not able to be varied and thus remains constant for every occurrence for the duration of simulations. Given the survival rate of tadpoles and juveniles is extremely low (0.01%), we modelled the impact of a draining event on the population by assuming the fecundity rate of breeders is reduced by 30% in that season. This value is based on expert opinion and observations made during the last 3 years of monitoring.

#### 2.3.3 Genetics

Genetic samples were taken from the *L. aurea* population during the two breeding seasons in 2020/21 and 2021/22. We used a 4 mm biopsy tool to collect skin samples from the rear toe webbing of sexually mature males and females. Skin samples were stored in vials containing 70% ethanol for preservation until analysis. Samples were sent to Diversity Arrays Technology (DART) Pty Ltd. (Canberra, Australia) for DNA extraction, as per Kilian et al. (2012). Single Nucleotide Polymorphism (SNP) genotyping and discovery by DART was conducted using the DArTseq protocol (Georges et al. 2018). This method involved employing PstL and Sphl restriction enzymes, custom proprietary bar‐coded adapters, PCR amplification, and sequencing using Illumina HiSeq2500. Sequences generated were processed using a proprietary DART analytical pipeline (Georges et al. 2018). SNPs were then filtered to ensure suitable read depth, reproducibility (> −99%) removal of monomorphic loci, call rate > 97%, removal of secondaries and no double sampling of individuals due to microchip loss using the DartR package (R Development Core Team 2022; Stock et al. 2023). We employed the “gl.subsample.loci” function from the Dart R package to randomly select 100 loci and then used “gl.alf” to report allele frequencies of the North Avoca population into Vortex. We used the default value for lethal equivalents (6.29) to define the effect of inbreeding on fecundity and survival. Our PVA modelled observed and expected heterozygosity, final number of alleles, final number of mt haplotypes and final number of lethal alleles/diploid.

#### 2.3.4 Management Alternatives

##### 2.3.4.1 Scenario 1: Status Quo

We modelled the population trajectory of the North Avoca population over the next 25 years assuming the Lagoon is drained at historical rates (82 events in 100 years) (hereafter referred to as the ‘*status quo*’ scenario). On these occasions we assumed the reproductive output of breeders was reduced by 30%.

##### 2.3.4.2 Scenario 2: Reducing the Frequency of Lagoon Draining

In scenario 2, we assumed that the incidence of a draining event in any given breeding season was halved; that is, the probability of a drainage event in any given year was reduced from 0.82 to 0.41. In this scenario, when a draining event occurred, the reproductive output of breeders was reduced by 30%, as estimated in the status quo scenario.

##### 2.3.4.3 Scenario 3: Creating an Additional Wetland

In Scenario 3, we assessed the impact of creating a new wetland adjacent to the Bareena wetland while the draining of the Lagoon continued at historical rates. We assumed the new wetland was positioned approximately 450 m away from the extant wetland, which is less than the long‐distance dispersal capacity of 1 km recorded for the species (Hamer et al. 2008). We assumed the constructed wetland was 20% of the area of the Bareena wetland and thus had a carrying capacity of 200 individuals. We also assumed that the new wetland was not connected hydrologically to the Lagoon and was initially unoccupied by frogs, relying on natural colonisation from the extant wetland. We assumed each individual had a 34% chance of dispersing between patches based on a published dispersal kernel for the closely related growling grass frog ( *Ranoidea raniformis* ) (Heard et al. 2012) and that there was no consequence of dispersal on survival.

##### 2.3.4.4 Scenario 4: Population Supplementation

In Scenario 4, we assumed that 10 adult individuals (5 males, 5 females) were added to the population every year while the Lagoon continued to be drained at historical rates. Translocated individuals were assumed to have the same genetic diversity that currently exists in the North Avoca population.

#### 2.3.5 Simulations and Sensitivity Analysis

We ran the four scenarios of our PVA each for 500 iterations to account for environmental and demographic stochasticity. At the end of the 25‐year forecast period, we recorded the probability of local extinction, population size, time to extinction, expected and observed heterozygosity, the final number of alleles, the final number of mt haplotypes and the final number of lethal alleles/diploid.

We conducted a sensitivity analysis to determine which model parameters had the greatest influence on predicted population size at the end of the 25‐year forecast period (Akçakaya and Sjögren‐Gulve 2000). We first ran our *status quo* model with and without genetics to explore the sensitivity of model predictions to inbreeding depression. We then ran the model by increasing and decreasing key parameters by 10% (initial population size, age‐specific mortality rates and fecundity) while holding all other values constant at their mean.

We also assessed the sensitivity of key model parameters in the management scenarios. For scenario 2 (reducing the frequency of lagoon draining), we ran additional scenarios where the incidence of lagoon draining varied from 0 (not occurring at all) to 1 (occurring every year) and where the effect of lagoon draining on reproductive output ranged from 0 (no reduction in reproductive output) to 100% (no egg/tadpole survival) (Appendix 2). For scenario 3 (creating an additional wetland), we ran scenarios where the carrying capacity of the new wetland was set to 100 and 300 individuals (Appendix 2). For scenario 4 (population supplementation), we varied the number of individuals translocated each year from 5 to 20 (Appendix 2).

## 3 Results

### 3.1 Scenario 1: Status Quo

**FIGURE 3:**
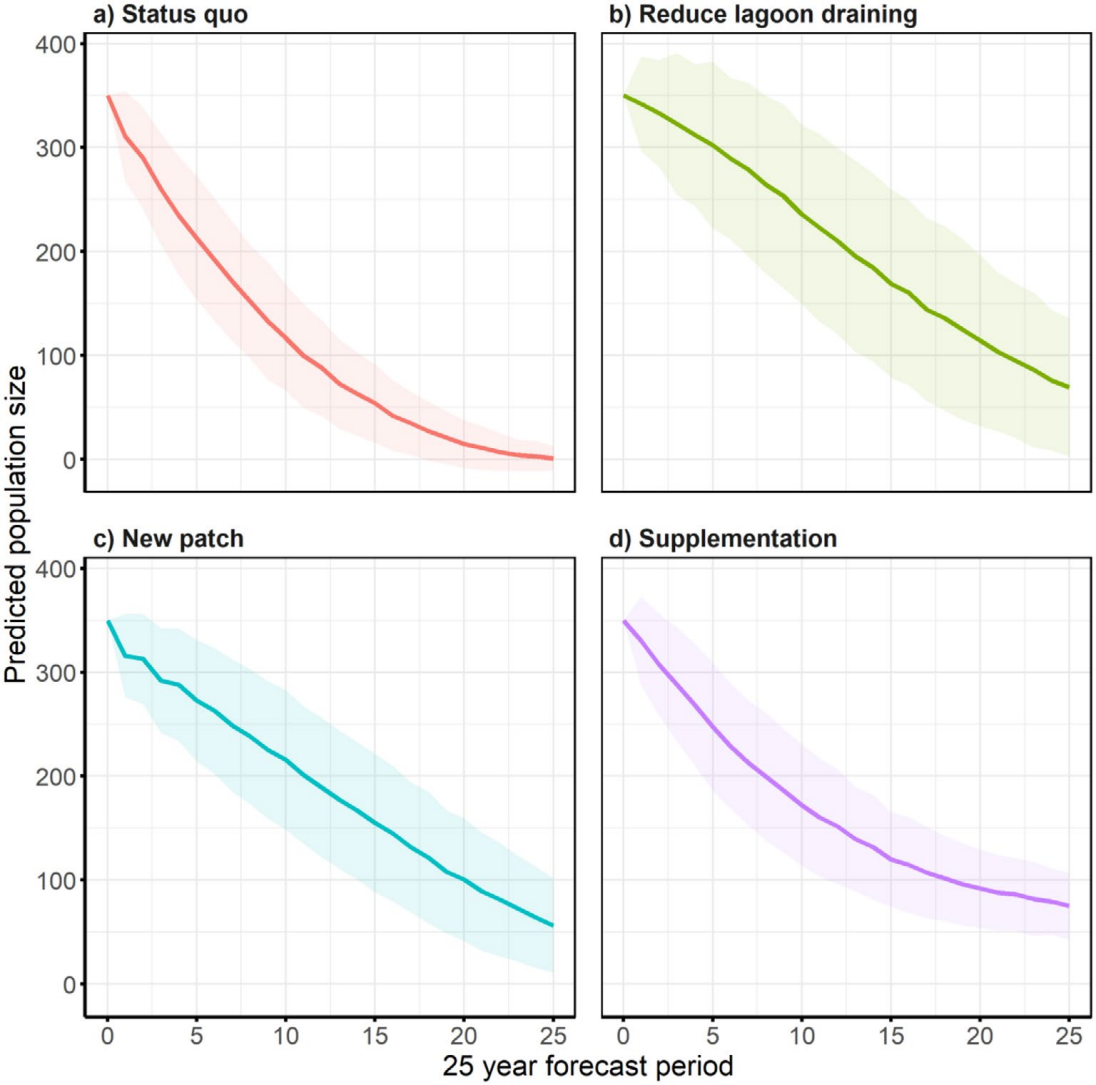
Predicted mean population size (solid lines) of the North Avoca *L.aurea* population over a 25 year forecast window under four management scenarios: (a) status quo; (b) reduce lagoon draining; (c) create additional wetland within dispersal distance, and (d) population supplementation. Shading indicates the standard deviation of the mean predicted population size.

**FIGURE 4:**
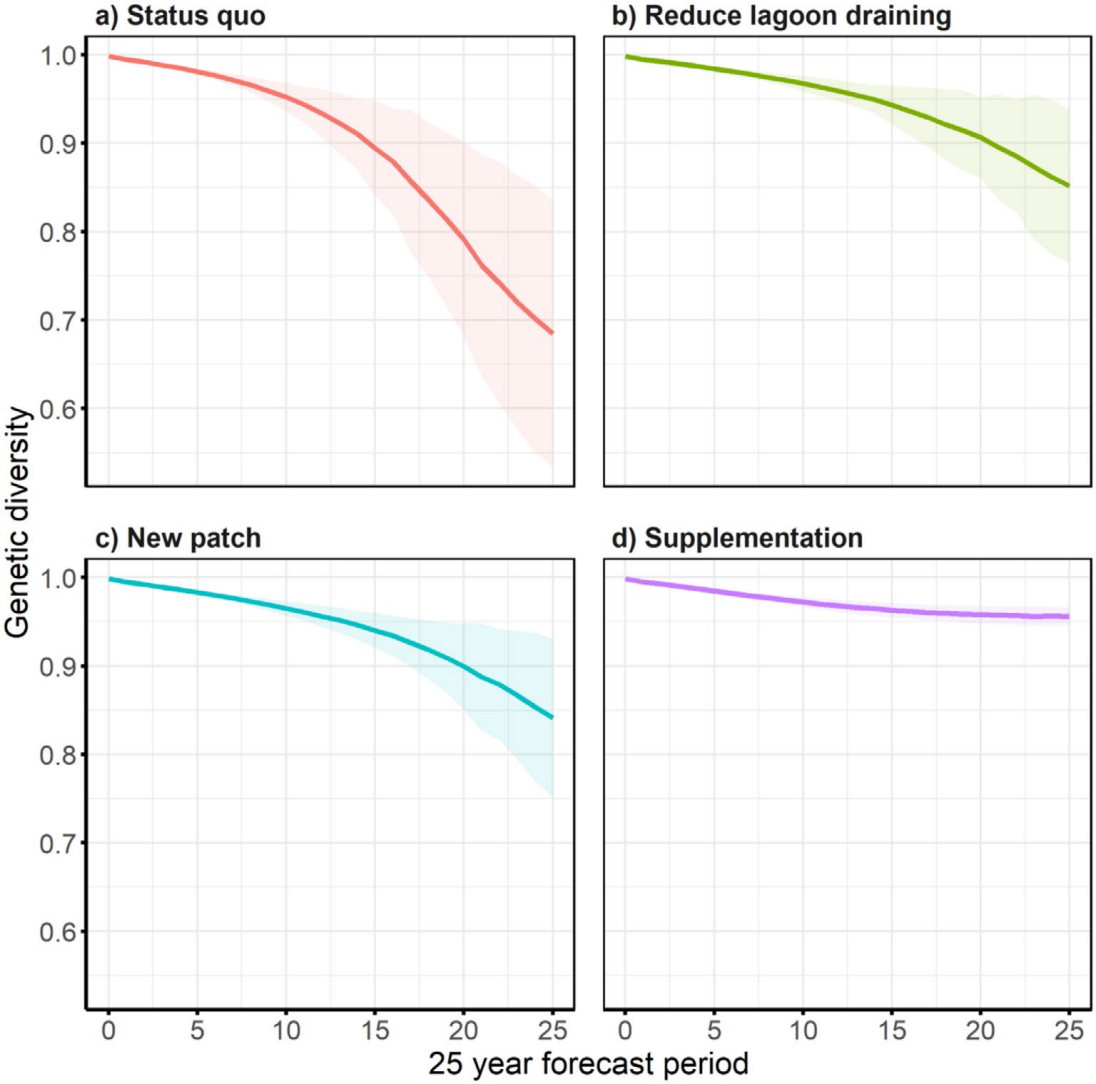
Expected genetic diversity (heterozygosity) of the North Avoca *L.aurea* population over a 25 year forecast window under for four management scenarios: (a) status quo; (b) reduce lagoon draining; (c) create additional wetland within dispersal distance, and (d) population supplementation. Shading indicates the standard deviation in heterozygosity.

Our PVA predicted a decline in the local *L. aurea* population under the status quo scenario when lagoon draining continued at historical rates. Under this scenario, population size declined from 350 individuals at the start of the simulation period to an average of six individuals (SD = 11.77) over a 25‐year forecast window (Figure 3). There was a 60% probability of local extinction within a mean time to extinction of 21.8 years (SD = 0.34). The final expected genetic diversity (expected heterozygosity) was on average estimated at 0.68 (SD = 0.15) (Figure 4), the final mean observed heterozygosity was 0.81 (SD = 0.19), and the final mean number of alleles was 5.54 (SD = 3.05). At the end of the 25‐year forecast window, the final number of mt haplotypes was 1.41 (SD = 0.49) and the final number of lethal alleles/diploid was 2.06 (SD = 0.92) (Table 2).

**TABLE 2:** Predicted heterozygosity, number of alleles, haplotypes and lethal alleles for the Bareena population in 25 years for each management alternative. Numbers in brackets are standard deviations.

Scenario	Expected heterozygosity	Observed heterozygosity	Final number of alleles	Final number of mt haplotypes	Final number of lethal alleles/diploid
1. Status quo	0.68 (0.15)	0.81 (0.19)	5.54 (3.05)	1.41 (0.49)	2.06 (0.92)
2. Reduced lagoon draining	0.85 (0.09)	0.90 (0.09)	14.44 (7.34)	1.82 (0.39)	2.35 (0.72)
3. Additional wetland	0.84 (0.09)	0.89 (0.10)	12.67 (5.56)	1.79 (0.40)	2.30 (0.73)
4. Population supplementation	0.96 (0.01)	0.98 (0.02)	52.98 (8.80)	2.00 (0)	2.97 (0.42)

### 3.2 Scenario 2: Reducing the Frequency of Lagoon Draining

Halving the frequency of lagoon draining resulted in the second highest predicted mean population size at the end of the 25‐year forecast horizon relative to the other scenarios tested. Under this scenario, the final mean population size after 25 years was 69 (SD = 66.30) individuals, compared to 6 individuals under the status quo scenario. The probability of local extinction was 6%, and the mean time to extinction was 23.03 years (SD = 2.10). The final expected genetic diversity (expected heterozygosity) was estimated at 0.85 (SD = 0.09) (Figure 4) and the final observed heterozygosity was 0.90 (SD = 0.09), while the final number of alleles was 14.44 (SD = 7.34). The final number of mt haplotypes was 1.82 (SD = 0.39) and the final number of lethal alleles/diploid was 2.35 (SD = 0.72).

### 3.3 Scenario 3: Creating an Additional Wetland

Creating a new wetland adjacent to the existing one ranked third in terms of estimated population size at the end of the 15‐year forecast period. Our PVA predicted that a mean of 56 (SD = 45.24) individuals will occupy both wetlands under this scenario. The probability of extinction was estimated at 6% and the mean time to extinction was 23.97 years (SD = 1.87). The final expected heterozygosity was predicted to be 0.84 (SD = 0.09) (Figure 4), the final observed heterozygosity was 0.89 (SD = 0.10) and the final number of alleles was 12.67 (SD = 5.56). In addition, the final number of mt haplotypes was 1.79 (SD = 0.40) and the final number of lethal alleles/diploid was 2.36 (SD = 0.73) (Table 2).

### 3.4 Scenario 4: Population Supplementation

Population supplementation as a management action ranked highest in terms of the mean predicted population size at the end of the simulation period. Under this scenario, the final population size was estimated to be 75 (SD = 32.08) individuals after 25 years. There were no instances where the population went extinct under this scenario given 10 individuals were released in the wetland each year. Population supplementation also ranked highest in terms of maintaining population genetic diversity; the final expected heterozygosity was estimated to be 0.96 (SD = 0.01) with relatively low uncertainty (Figure 4), the final observed heterozygosity was 0.98 (SD = 0.02) and the final number of alleles was 52.98 (SD = 8.80). Lastly, the final number of mt haplotypes was 2.00 (SD = 0) and the final number of lethal alleles/diploid was 2.97 (SD = 0.42) (Table 2).

### 3.5 Simulations and Sensitivity Analysis

**FIGURE 5:**
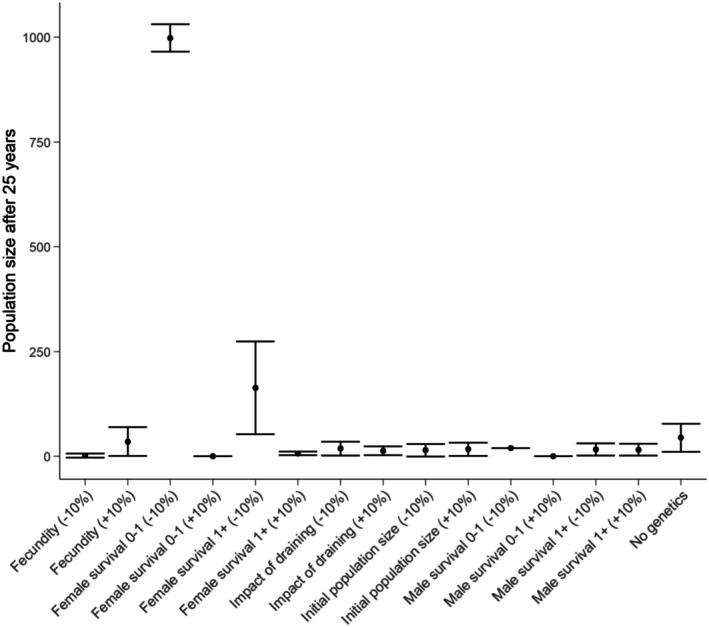
The predicted population size at the end of the 25‐year forecast period under the status quo scenario (82% probability of lagoon draining in any breeding season) when key parameters are increased and decreased by 10%.

The most sensitive parameter in the *status quo* model was female mortality at 0–1 years (eggs, juveniles and metamorphlings) (Figure 5). Decreasing mortality of this sex/age class by 10% resulted in the final population size approaching the pre‐defined carrying capacity of 1000 individuals. Female mortality of individuals aged 1–2 also had an effect, with an average of 177 individuals expected to survive after 25 years when this parameter was reduced by 10%.

Reducing the mortality of males aged 1–2 years by 10% also increased the final population size. Finally, running the PVA without genetic processes resulted in an average size of the final population being 37 individuals, up from the 6 individuals predicted when genetic processes were included (*status quo* scenario). In contrast, the initial population size and fecundity had relatively little influence on population dynamics (Figure 5).

Additional sensitivity analyses of scenarios 2, 3 and 4 provided greater context to the potential magnitude of impact associated with management actions. Varying the impact of lagoon draining on reproductive output in scenario 2 from 0 (no occurrence) to 1 (occurring every year) demonstrated that the population was highly sensitive to even modest increases in stress on reproductive output. Simulations predicted that the final population would be reduced to small numbers even when lagoon draining negatively impacted reproductive output by less than 10% (Figure S2.1, Appendix 2). Increasing the carrying capacity of the created wetland in scenario 3 from 100 to 300 individuals positively influenced final population size, as did increasing the number of individuals supplementing the extant population in any given year from 5 to 20 (scenario 4) (Figure S2.2, Appendix 2).

## 4 Discussion

Small, isolated populations that occupy human‐mediated coastal zone processes are increasingly susceptible to local extinction from catastrophic events, demographic stochasticity and land use change. In this study, we developed a PVA for an isolated population of the endangered *L. aurea* in south‐east Australia to predict extinction risk over a 25‐year forecast window. We incorporated key ecological, demographic and genetic processes into our model to assess extinction risk under four management alternatives. Our PVA predicts that if the Lagoon continues to be drained at historical rates, the current population of 350 individuals has a 60% chance of local extinction over the 25‐year period, suggesting that management is needed to prevent local extinction. Supplementing the population each year with individuals from elsewhere ranked highest in terms of the final population size, followed by halving how often the Lagoon is drained and creating a new wetland, although uncertainty around each scenario was high. Our sensitivity analysis also demonstrated that the future size of the *L. aurea* population is strongly influenced by the survival of females in the 0–1‐year stage when eggs turn into tadpoles and pre‐metamorphic frogs. Survival of this life stage of biphasic amphibians relies heavily on the hydroperiod of waterbodies to allow their successful transition to a land‐ based lifestyle (Nolan et al. 2023). This suggests that addressing the underlying ecological processes of population viability is of paramount importance when determining the appropriate conservation strategies for dealing with environmental change.

### 4.1 Lagoon Draining

Under the status quo scenario, our PVA predicted that the *L. aurea* population will reduce from a starting population size of 350 individuals to an average of 6 individuals (SD = 11.77) after 25 years, with a 60% probability of extinction. Extinction risk is high because lagoon draining results in the desiccation of eggs and tadpoles as the wetland dries and renders this breeding habitat unavailable until the wetland recharges. Halving the frequency of lagoon draining during the *L. aurea* breeding season was an effective management action, reducing the probability of extinction from 60% to 6% and increasing the mean final population size to 69 (SD = 66.30) individuals.

However, this result was underpinned by our assumption that a draining event reduced the reproductive output of all breeding pairs in the population by 30%. While this estimate was based on anecdotal observations of the population over the last 3 years, it is highly uncertain. To explore this uncertainty further, we ran additional scenarios where the impact of lagoon draining on reproductive output ranged from 0% to 100%. This analysis indicated that the impact of lagoon draining on reproductive output only has to be relatively small (< 10%) for the final population to be reduced to small numbers. This makes sense as artificial lagoon draining is more likely to occur immediately following heavy rainfall, where flooding risk for surrounding urban areas is greatest. On the Central Coast of NSW this occurs typically in late summer and early autumn (NSW Department of Primary Industries, n.d.), which coincides with the breeding season of this threatened amphibian. As such the risk of lagoon draining occurring after an *L. aurea* breeding event is relatively high.

Our PVA predicts that the underlying ecological processes of population viability for *L. aurea* at Avoca Lagoon are reproduction (successful breeding) and age‐specific survival between 0 and 1 years. Wetlands must thus contain some water (> 10 cm for a minimum of 12 weeks) during the breeding season for offspring to develop, reach metamorphosis and transition to a land lifestyle (Christy 2001; Davis et al. 2019; Hamer et al. 2008; Pickett et al. 2016). Any draining event that dries the lagoon within the breeding season drives mortality of eggs and tadpoles in the wetland, effectively reducing recruitment of offspring into the adult population.

While halving the frequency of artificial lagoon draining events is an effective conservation strategy for *L. aurea* at Avoca Lagoon, it also requires balancing hydrological management to prevent flooding of adjacent infrastructure. A recent investigation of the hydrogeological interactions between groundwater and surface water in Avoca Lagoon demonstrated that maintenance of hydroperiod and water quality conditions suitable for *L. aurea* breeding could be achieved while also balancing flood risk (Palombi et al. 2025). The study showed that lagoon draining disrupts groundwater discharge that is critical for wetland saturation and that engineered overflow solutions could achieve flood mitigation while protecting *L. aurea* habitat. This includes the installation of drainage channels or storage basins to manage extreme rainfall runoff without requiring full lagoon draining (Palombi et al. 2025). The implications of such an approach on population viability are unknown and further modelling combining a PVA with this hydrological model are required to compare the relative probability of local extinctions between this proposed solution and those already investigated in this study.

### 4.2 Creating New Habitat

Habitat creation projects have worked in several locations in Australia to support the expansion of *L. aurea* populations (Pickett et al. 2013; Valdez et al. 2019; Gould, Beranek, et al. 2024; Gould, Callen, et al. 2024), with an emphasis on creating a habitat mosaic that reduces the prevalence of threats and meets the needs of both life stages of biphasic amphibians (Nolan et al. 2023; Gould, Beranek, et al. 2024; Gould, Callen, et al. 2024). In our PVA, creating new habitat adjacent to the existing wetland ranked third out of the four scenarios tested, with a mean predicted final population of 56 individuals (SD = 45.24) after 25 years. Although this scenario did not perform as well as population supplementation or halving the incidence of lagoon draining in terms of final population size, it did reduce the probability of extinction to 6%, which is significantly less than the status quo scenario (60%). This scenario improved the persistence of the *L. aurea* population because we assumed the new wetland was not hydrologically connected to the Lagoon and so retained water throughout the breeding season when drainage events occurred and because it was within dispersal distance from the Bareena wetland. Additional sensitivity analyses showed that the final mean population size was influenced by the carrying capacity of the new patch, so further gains in population size could be achieved if the size of the new patch could be increased.

Habitat creation projects have been established for *L. aurea* populations in coastal NSW and guidelines have been developed for their design (Pyke and White 1996). For example, new ponds were created for *L. aurea* at Sydney Olympic Park to offset urbanisation in the 1990s. Monitoring of these populations has shown that the new habitat has been successfully and naturally colonised by individuals, but overall densities were lower than those observed in historical habitat, suggesting that the offset habitats may be less functional for the species than the original habitat (Pickett et al. 2013). New ponds for *L. aurea* have also been created on Kooragang Island, near Newcastle, to increase the number of sites available for reproduction and connectivity and also to provide additional aquatic refuges, which have also been shown to be successful in terms of natural colonisation (Klop‐Toker et al. 2021; Gould, Beranek, et al. 2024; Gould, Callen, et al. 2024). Outside of these examples, historical management efforts surrounding habitat creation, translocation and reintroduction of *L. aurea* have often been unsuccessful (White and Pyke 2008) possibly because they did not appropriately address disease impacts through habitat manipulations (Scheele et al. 2021) or new habitat was not selected by adults for breeding (Valdez et al. 2019). As such, caution must be applied when designing additional habitat for the conservation of a population and in some cases a greater benefit may arise by focusing management efforts on protecting or improving the existing habitats rather than creating new ones (Southwell et al. 2016; Simpkins et al. 2022).

### 4.3 Population Supplementation

Supplementing the wetland with a relatively small cohort of 10 individuals each year was the highest‐ranked management scenario tested, with a mean final population size of 75 individuals (SD = 32.08) at the end of the 25‐year forecast period. An additional benefit of this scenario over the alternatives is that it resulted in the maintenance of the highest level of genetic diversity throughout the simulation period, with the final expected heterozygosity estimated at 0.96 (SD = 0.01). Predictions of genetic diversity were also relatively stable and certain over the forecast period compared to the other management alternatives tested. This was due to the regular introduction of individuals with high genetic diversity each year from nearby populations. Not surprisingly, the mean final population size under this scenario was sensitive to the number of individuals translocated; the final population size increased as the number of individuals supplemented increased from 5 to 20 per year (Appendix 2).

Although the population supplementation was the highest ranked scenario in terms of mean final population size and genetic diversity, there are several drawbacks to this approach. Firstly, it incurs ongoing costs that will continue indefinitely and does not directly address major threats to the population, in particular the threat of lagoon draining on offspring. Thus, any delays in the release of individuals could initiate a new population decline. Secondly, population supplementation requires a constant source of individuals for translocation. In this case, individuals could be sourced from nearby populations in Sydney or Newcastle which are about 90 km away. However, these populations fluctuate significantly between breeding seasons and might also be at risk of local extinction themselves, placing further strain on them if reproductively active adults are being constantly removed. Additionally, we assumed the survival rates of translocated individuals were identical to those occupying the wetland; however, recent research suggests supplemented individuals might have much lower rates of survival in the first few years after a translocation as they acclimatise to the local conditions (Gould et al. 2023).

While a range of genetic rescue strategies exists to support small populations, most of these focus on supplementation (e.g., the deliberate movement of individuals between populations). Such approaches require ongoing sustained effort for the North Avoca population because the lagoon draining threat to population viability would continue to exist. De‐extinction initiatives (e.g., cloning, back‐breeding and genetic engineering) would also be costly and require a number of generations to be tracked before confirming this as a suitable approach. A more sustainable but unattainable genetic rescue strategy is gene flow restoration—the reconnection of fragmented populations. While we have undertaken gene flow restoration for the green and golden bell frog and other threatened amphibians, this works best in non‐urbanised areas. The reconnection of fragmented populations in urban areas has the potential to expose moving individuals to a range of additional threats such as road mortality and predation by pets. Thus, supplementation is currently likely to be the most cost‐effective genetic rescue strategy for retaining the viability of the North Avoca population.

### 4.4 Genetics

Our PVA focused on assessing management actions that might reduce local extinction risk and improve genetic diversity, which are principles consistent with global conservation strategies for amphibians (Pabijan et al. 2020). However, genetic diversity is often overlooked in many models that focus on extinction rates and demographic stochasticity, even though it is critical in assessing the health and resilience of populations (Pierson et al. 2015; Auffarth et al. 2017). Despite its power, only a minority of PVA studies incorporate genetic processes, with most relying entirely on demographic data (Chaudhary and Oli 2020). The inclusion of genetic data for amphibian PVAs is even less common (Shaffer et al. 2015; Kosch et al. 2024). Integrating genetic data allows PVAs to account for processes such as inbreeding depression, loss of heterozygosity and genetic drift, all of which are critical for small, isolated populations. By modeling genetic variation alongside demographic and environmental factors, these models can more accurately predict extinction risk and evaluate the effectiveness of management actions aimed at maintaining or enhancing genetic diversity (Reed et al. 2002; Auffarth et al. 2017; Stock et al. 2023).

Our sensitivity analysis of the status quo scenario modelled with and without genetic processes demonstrates the benefit of including such information in PVAs for small, fragmented populations. The incorporation of genetic data in our PVA revealed moderate levels of heterozygosity and allele diversity in the North Avoca population of *L. aurea* . Genetic diversity was predicted to decline under the status quo lagoon draining scenario over 25 years. This is a critical finding showing that inbreeding contributes to elevated local extinction risk. Overall, the genetic analysis emphasises the need for proactive management strategies that address not only demographic and environmental pressures but also the genetic health of the population to ensure long‐term viability. For example, population supplementation was shown to mitigate genetic decline, but so was the more passive conservation action of halving the frequency of lagoon draining. Considering extinction risk and genetic health via the more widespread adoption of genetic monitoring in conservation planning may ensure that management strategies prioritise maintaining or enhancing genetic diversity.

### 4.5 Limitations and Further Research

In our PVA, the effect of lagoon draining on the *L. aurea* population was modelled by specifying the chance of a draining event occurring in any given year and the effect this has on reproductive output. However, female *L. aurea* are prolonged breeders, with only a portion of mature females engaged in breeding at a given time during the breeding season (Christy 2001). Therefore, the impact of lagoon draining on reproductive output will be highly variable, depending on the duration and number of dry periods, and when the lagoon is drained in the breeding season. The longer the wetland remains without sufficient water for reproduction, the more likely breeding will not be achieved in that season. If this occurs early in the breeding season or the dry period is short in duration, breeding may resume at full capacity when conditions are once again suitable. If wetland drying occurs late in the breeding season or the dry period is long, it may extend past the end of ideal breeding conditions for the species. Thus, our model could be further developed to incorporate more detailed scenarios of lagoon draining and its effect on the population. This includes hydrological controls on water salinity and quality which were, at the time of conducting this PVA, poorly understood (Palombi et al. 2025).

We found that predictions of population size were most sensitive to female mortality in the pre–metamorph stage (0–1 years). Decreasing this parameter by 10% resulted in the population persisting at close to carrying capacity over the next 25 years. Survival of females in the next age class (1–2 years) was the next most sensitive parameter; a decrease in this parameter by 10% resulted in a mean final population size of 220 individuals. Similarly, male mortality of 1–2 year olds ranked as the third most sensitive parameter. This result is not surprising as larval and juvenile mortality is often identified as the most sensitive parameter in amphibian PVAs (Biek et al. 2002; Hamer and Mahony 2007; Zambrano et al. 2007; Pickett et al. 2016), however we acknowledge that despite using the best available estimates, this is a potential source of inaccuracy. Given we parameterised our model based on the existing literature, future research could conduct experimental studies on the *L. aurea* population to measure tadpole and juvenile survival both in the presence and absence of lagoon draining. Mark‐recapture studies are difficult for tadpoles because they are difficult to uniquely mark and the large area of the wetland means that a very large number of tadpoles would have to be captured (McFadden et al. 2008). However, fenced or basket enclosures could be created in the wetland containing a known number of tadpoles, with survival monitored over time under different environmental conditions. Either way, given our objective was to compare the relative probability of local extinction of the population between modelled scenarios, this limitation does not change the relative trends in the outputs as the parameters were consistently applied.

## 5 Conclusion

While coastal regions support highly biodiverse ecosystems that are considered the most valuable biome globally on a per‐hectare economic value basis (Barbier et al. 2011; Costanza et al. 2014), these regions are also the most densely populated and more than 50% of the world's coastal systems are recognised as degraded as a result of this high human activity (Williams et al. 2022). We demonstrate that ongoing manipulations of coastal systems to protect the built environment from flooding events have the capacity to drive threatened freshwater organisms to local extinction. Our study demonstrates coastal zone modifications can be a key threat to organisms of coastal freshwater wetlands that rely on a stable hydroperiod for successful reproduction. With only 15% of the world's coastlines retaining their ecological integrity, the imperilled global biodiversity that exists in this dynamic environment requires a nuanced conservation approach that preserves both built and natural assets into the future.

Our PVA demonstrated that reducing lagoon draining frequency, creating a new patch or population supplementation may reduce the extinction risk of the North Avoca *L. aurea* population. Of the scenarios tested, reducing the frequency of lagoon draining avoids the logistical challenges and long‐term costs of population supplementation and patch creation while addressing the root cause of population decline associated with the loss of breeding habitat. There is no doubt there is a need to balance flood‐risk mitigation with ecological needs (retaining water during breeding seasons) which might require additional engineering and construction effort, but it offers the best balance of ecological benefit, cost‐effectiveness and feasibility of the management actions assessed here. Such an approach may serve as a model for other freshwater‐dependent biodiversity threatened by similar hydrological management practices globally.

## Author Contributions

**Alex Callen:** conceptualization (equal), data curation (equal), formal analysis (equal), funding acquisition (lead), investigation (equal), methodology (equal), project administration (lead), resources (lead), supervision (equal), writing – original draft (equal), writing – review and editing (equal). **Heather Maher:** data curation (lead), formal analysis (equal), investigation (lead), methodology (equal), validation (equal), visualization (equal), writing – original draft (lead), writing – review and editing (equal). **John Gould:** data curation (supporting), formal analysis (supporting), methodology (supporting), validation (supporting), writing – review and editing (supporting). **Matt W. Hayward:** conceptualization (supporting), funding acquisition (supporting), methodology (supporting), project administration (equal), resources (equal), writing – review and editing (equal). **Samantha Sanders:** investigation (equal), writing – review and editing (equal). **Michael Mahony:** conceptualization (supporting), funding acquisition (equal), resources (supporting), writing – review and editing (equal). **Gabriel C. Rau:** resources (supporting), validation (supporting), writing – review and editing (equal). **Sarah Stock:** data curation (supporting), formal analysis (supporting), methodology (equal), software (lead), validation (supporting), writing – review and editing (equal). **Kate Tunstill:** investigation (supporting), validation (supporting), writing – review and editing (supporting). **Darren M. Southwell:** conceptualization (equal), data curation (equal), formal analysis (lead), methodology (lead), supervision (lead), validation (lead), visualization (lead), writing – original draft (equal), writing – review and editing (lead).

## Funding

This work was supported by Central Coast Council.

## Conflicts of Interest

The authors declare no conflicts of interest.

## Supporting information


**Appendix S1:** Supporting Information.

## Data Availability

The modelling parameters for this study are outlined within the document. The PVA parameters can be found in Table 1, while the population estimate model outputs are in the Appendix. The data supporting the findings of this study are openly available in Figshare at https://doi.org/10.6084/m9.figshare.30489296.
