# Supplementary material for: Looking Forward: Evaluating Management Scenarios for an Isolated Amphibian Population in a Dynamic Coastal Environment
Source: Ecol Evol. 2026 Jan 12;16(1):e72598. doi: 10.1002/ece3.72598 (PMC12796506; doi:10.1002/ece3.72598)
Supplement: Supplementary file 1 — Appendix S1: Supporting Information. [file ECE3-16-e72598-s001.docx]

**Appendix 1: Estimating population size**

Population size estimates and survival of green and golden bell frogs at Bareena was conducted using capture-recapture data with POPAN models. The analysis was conducted over 18 sampling events, aggregated by month, and only including months in the breeding season of 2020 and 2023. Estimates were derived from 502 individually tagged frogs with accurate capture data. These models investigate whether survival and the probability of being captured, as well as the probability of entering the population are time dependent or time independent (eg- in frogs, the probability of being detected may be influenced by time due to air temperature differences across the seasons).

Eight candidate models were considered in the analysis and were a combination of the following model types:

1. POPAN with time-dependent survival (phi), capture (p) and entrance (pent) probabilities.
2. POPAN with time-independent survival, capture and entrance probabilities.
3. POPAN with varying time-dependent and time-independent survival, capture and entrance probabilities.

Models were compared using the Akaike Information Criterion (AIC), and the model selected as the best representation of the real system was the one with the lowest AIC value (Table 1). The POPAN model that best explained the population parameters based on green and golden bell frog capture-recapture data collected at North Avoca indicated that survival between survey periods is constant (time-independent), but that capture probability varies with time. The probability of entering the population was also dependent upon time, which is biologically plausible, given that adults come to Bareena Wetland for the breeding season and then disperse away again. The model estimated apparent survival between capture events (surveys) as 0.86 (95% CI: 0.84 -0.89), suggesting a high rate of survival of individuals between surveys. The model also indicated detection probability varied with time (range) with a low estimate of 0.19 (95% CI: 0.17 – 0.22), and a pent estimate of 0.049 (births and immigrations). So while survival between captures is high, individuals are not easily detected (low rates of detection) and additions to the population via births and immigration (pent) are time varying but very small.

Table 1 AICc values for candidate POPAN models with variations of time-dependent and time-independent parameters for survival, capture probability and entrance probability. The first model is the best candidate model, having the lowest AICc.

| **Model** | **Npar** | **AICc** | **DeltaAICc** | **Weight** | **Deviance** |
| --- | --- | --- | --- | --- | --- |
| BEST MODEL: Survival (time-independent), detection probability (time-dependent), entrance probability (time-dependent) | 37 | 1838.219 | 0.0000 | 0.9989 | -1793.487 |
| Survival (time-dependent), detection probability (time-dependent), entrance probability (time-dependent) | 53 | 1847.191 | 8.972 | 0.0111 | -1820.418 |
| Survival (time-dependent), detection probability (time-dependent), entrance probability (time-independent) | 37 | 1914.808 | 76.589 | 0.0000 | -1716.898 |
| Survival (time-independent), detection probability (time-dependent), entrance probability (time-independent) | 21 | 1919.528 | 81.309 | 0.0000 | -1677.724 |
| Survival (time-dependent), detection probability (time-independent), entrance probability (time-dependent) | 36 | 1988.371 | 150.152 | 0.0000 | -1641.140 |
| Survival (time-independent), detection probability (time-independent), entrance probability (time-dependent) | 20 | 2068.754 | 230.534 | 0.0000 | -1526.391 |
| Survival (time-dependent), detection probability (time-independent), entrance probability (time-independent) | 20 | 2342.247 | 504.028 | 0.0000 | -1252.898 |
| Survival (time-independent), detection probability (time-independent), entrance probability (time-independent) | 4 | 2491.867 | 653.648 | 0.0000 | -1070.270 |

Because POPAN models do not derive a direct estimate of population size, they need to be derived using model estimates.The POPAN model also calculates “N”, the superpopulation, or the total number of individuals that enter the sampled population between the first and last survey occasion. The superpopulation for the best model was 874 individuals, representing the total number of individuals that ever entered the North Avoca survey footprint between the first survey in September 2020 and the last survey in March 2023. This may appear a high population estimate, when compared with the capture event model estimates of abundance but this is spread over three seasons where monthly survival estimates vary between 100 and 300 with confidence intervals as high as 680. Mean seasonal estimates are significantly different from each other in every year – 181 in 2020-2021, 260 in 2021-2022 and 301 in 2022-2023 (Anova, F(2, 14) = (44.21), p = <0.00 and Tukey-Kramer’s HSD (q scores less than q critical level of 3.701). Additionally, low capture probabilities erode the confidence of the accuracy of the estimates suggesting this population estimate is of medium accuracy. Therefore, a conservative population estimate of 350 individuals is considered appropriate for the survey area for the North Avoca population of green and golden bell frog.

**Appendix 2: Additional sensitivity analysis**

We assessed the sensitivity of key model parameters in the management scenarios. For scenario 2, we ran additional scenarios where the incidence of lagoon draining varied from 0 (not occurring at all) to 1 (occurring every year) and where the effect of lagoon draining on reproductive output ranged from 0 (no reduction in reproductive output) to 100% (no egg/tadpole survival) (Figure S2.1). For scenario 3, we ran scenarios where the carrying capacity of the new wetland was set to 100 and 300 individuals (Figure S2.2). For scenario 4, we varied the number of individuals translocated each year from 5-20 (Figure S2.2). We ran the four scenarios of our PVA each for 100 iterations to account for environmental and demographic stochasticity. At the end of the 25 year forecast period, we recorded the probability of local extinction, population size, time to extinction, expected and observed heterozygosity, the final number of alleles, the final number of mt haplotypes and the final number of lethal alleles/diploid.


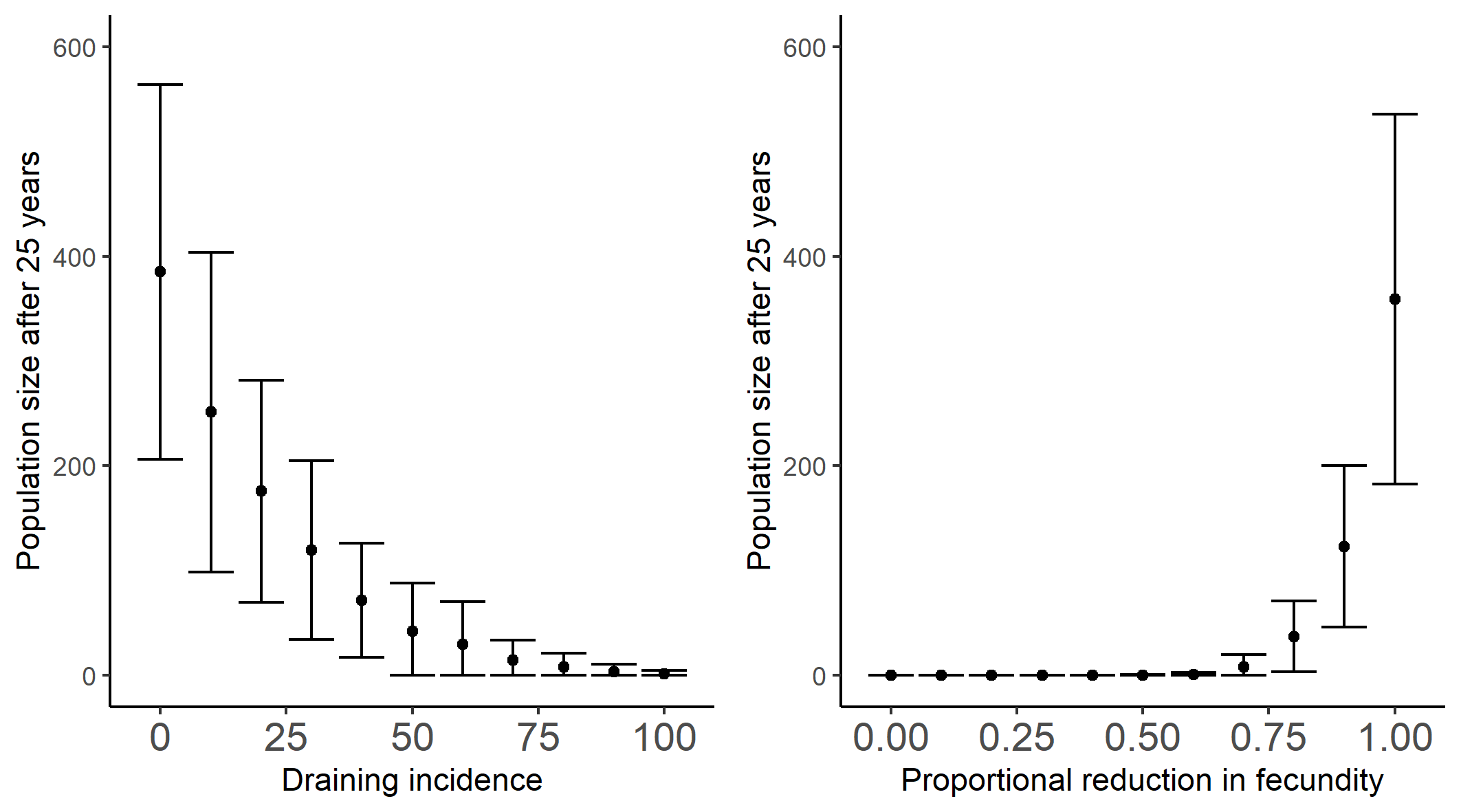


Figure S2.1: Predicted population size at the end of the 25 year forecast period when the incidence of lagoon draining is varied from 0 to 100% chance in any given year (left) and when the proportional reduction in survival is varied from 0 to 1 (right) (Scenario 2).


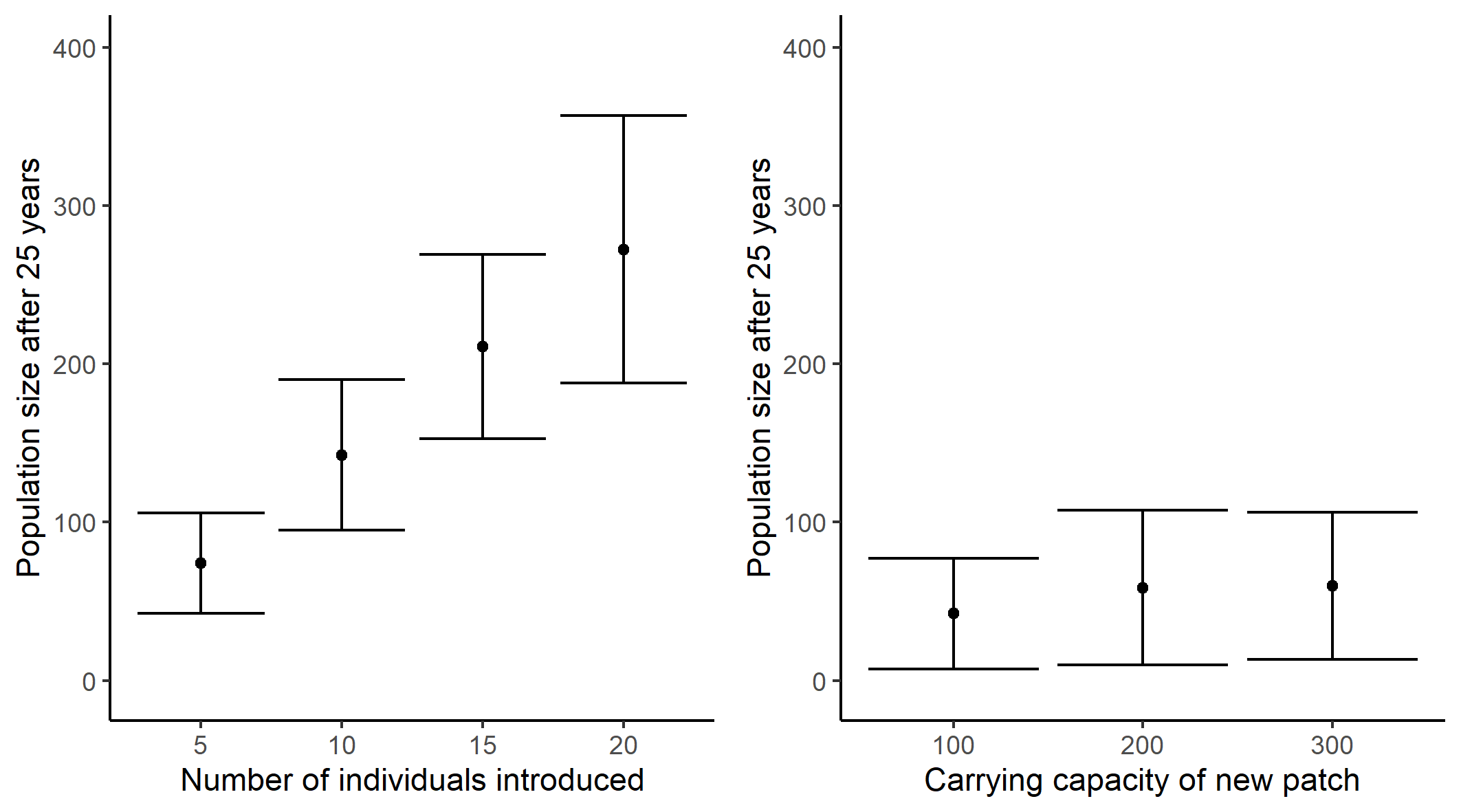


Figure S2.2: The predicted population size at the end of the 25 year forecast period when the number of individuals translocated to the wetland is increased from 5 to 20 per year (left) (Scenario 4) and when the carrying capacity of the new patch is varied from 100 to 300 individuals (right) (Scenario 3).
